# A novel low-power laser-mediated transfer of foreign molecules into cells

**DOI:** 10.1038/srep22055

**Published:** 2016-02-23

**Authors:** Shigehiko Yumura

**Affiliations:** 1 Department of Functional Molecular Biology, Graduate School of Medicine, Yamaguchi University, Yamaguchi 753-8512, Japan

## Abstract

Efficiently introducing molecules such as chemical drugs, proteins, or nucleic acids into cells is a central technique in cell and molecular biology, gene therapy and regenerative medicine. The cell membrane is a critical barrier for this purpose. While many approaches exist, some of which are applicable to single cells that researchers specify under microscopy, no reliable and efficient technique has been invented. In this study, cells were cultured on a coverslip that had been coated with carbon by vapor deposition, and a laser beam was focused on a small local spot beneath a single cell under microscopy. The absorbed energy of the laser beam by the carbon made a pore only in the cell membrane that was attached to the carbon coat, which resulted in an efficient introduction. An inexpensive and lower-power laser could be used for this method, and the introduction efficiency was 100% without any loss of cell viability. This new technique will provide a powerful tool not only to research but also to many applied fields.

Efficiently introducing molecules such as chemical drugs, proteins, or nucleic acids into cells is a central technique in cell biology, molecular biology, gene therapy and regenerative medicine. The cell membrane is a critical barrier for this purpose. There are many physical, chemical, and biological approaches, such as microinjection, electroporation, lipofection, and virus-mediated transfection^1^,^2^,^3^. Chemical and biological methods have been frequently used due to their high introduction efficiencies, but these methods cannot be used for therapeutic purposes for humans because harmful chemicals or viruses may remain in the transfected cells. Physical methods are ideal for this purpose. Some of these techniques are applicable to single cells that researchers specify using microscopy, but no reliable and efficient technique has been invented. A high-power femtosecond laser beam has been shown to make a pore in the cell membrane and introduce foreign molecules into cells when they exist in an external solution^4^; however, the laser beam in this method cannot be properly focused only on the cell membrane, which often results in intracellular structure disorganization and cellular rupture. In the present study, we overcame this problem. A coverslip was coated with carbon by vapor deposition. When the laser beam was focused on a small local spot beneath the cell under microscopy, the absorbed energy of the laser beam by the carbon made a pore only in the cell membrane that was attached to the carbon coat, resulting in efficient introduction. The wound pores were immediately closed by the cell wound repair system without leaving any damages. An inexpensive and lower-powered laser could be used for this method, and the introduction efficiency was 100%. In addition, the minimum volume of the external solution containing foreign molecules was only 10 μl, which eliminates the cost of valuable molecules such as fluorescent probes or expensive drugs. The total time required for the operation was only 5 min. This new technique will provide a powerful tool not only to research but also to many applied fields.

## Results

**Figure 1:**
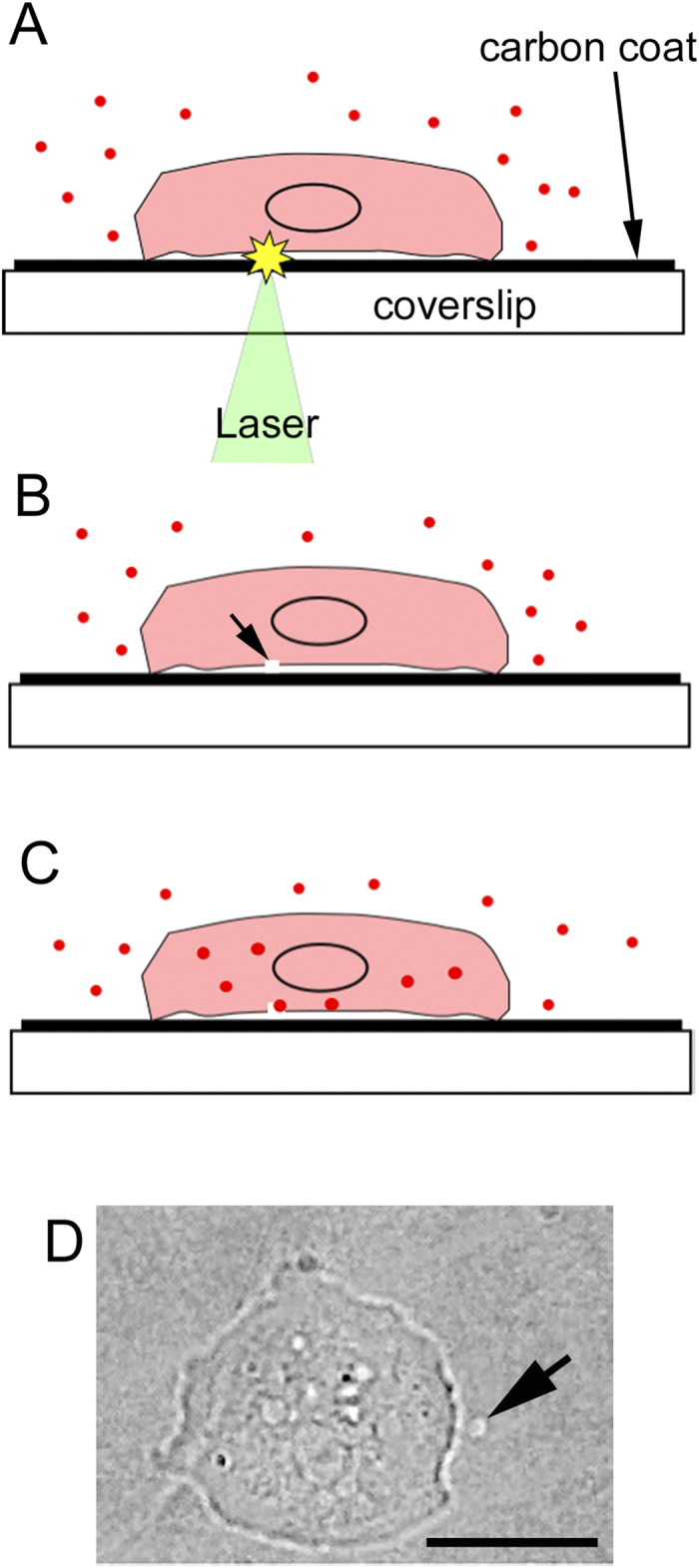
The principle for the new laser poration method. The surface of a coverslip was coated with carbon by vapor deposition with 20 nm thickness. (**A**) Under microscopy, a laser beam was focused on a small local of the carbon coat underneath the cell. (**B**) The energy of the laser beam was absorbed by the carbon and made a pore only in the ventral cell membrane (arrow). (**C**) The foreign molecules (red dots) in the external solution entered the cell through the pore. The pore was immediately recovered by the cell wound repair system without leaving any damage. (**D**) When the laser beam was focused on a small local spot of the carbon coat near the cell, the carbon coat was peeled off, appearing as a small white spot (arrow). Because the laser power used for the actual laser poration was much weaker, the carbon coat was not peeled off. Bar, 10 μm.

For the new laser poration technique, a nanosecond pulse laser was directed toward the sample through an objective of an inverted fluorescence or total internal reflection fluorescence (TIRF) microscope (Fig. 1A). The coverslip of the glass-bottom chamber was coated with carbon by vapor deposition with 20 nm thickness. When the laser beam was focused on the surface of coverslip, the carbon coat was peeled off, appearing as a small white spot (Fig. 1B). After the focus was set on the surface of the coverslip in this way, the laser beam was applied to the cells attached to the coverslip.

**Figure 2:**
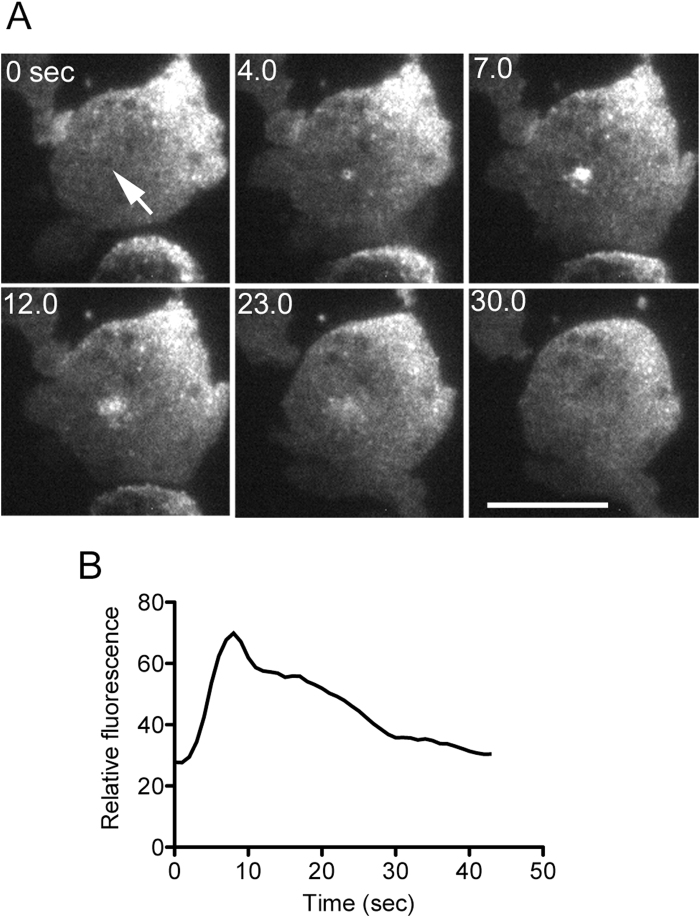
Wound repair after laser poration. (**A**) A laser beam was applied at a small spot (arrow) on *Dictyostelium* cells expressing GFP-lifeact for 10 msec under TIRF microscopy. Bars, 10 μm. (**B**) This figure shows the time course of fluorescence intensity at the wounded spot.

Previously, we showed that when *Dictyostelium* cells expressing GFP-lifeact, a marker for actin filaments, were injured by a microneedle under fluorescence microscopy, actin transiently accumulated at the wound site^5^. A laser beam was applied at a small spot (less than 0.5 μm in diameter) of cells expressing GFP-lifeact for 10 msec. Fig. 2B shows a time course of fluorescence intensity indicating that the cells were wounded by the laser application (Fig. 2). The actin accumulation occurred at the laser-spotted area only in the ventral cell membrane, which was confirmed by TIRF microscopy. Therefore, the pore in the ventral cell membrane was wounded upon the application of the laser beam. The low-power laser used here was not able to induce a wound if the coverslip was not coated with carbon.

**Figure 3:**
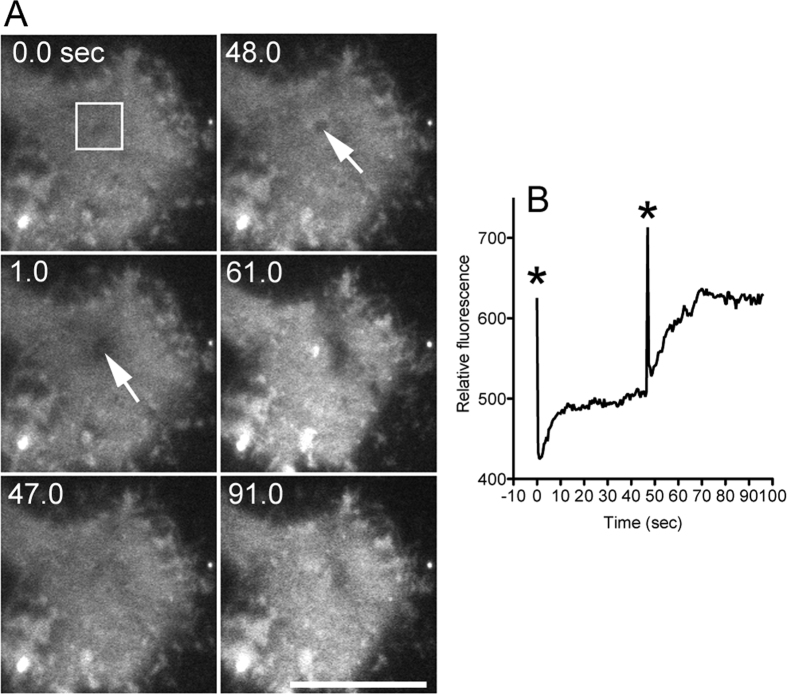
Introduction of a fluorescent dye into Cos-1 cells. (**A**) A laser beam was applied two times to Cos-1 cells in the presence of 10 μM FM1-43 in the external solution. The arrows show the position of the laser application. Bars, 10 μm. (**B**) This figure shows the time course of fluorescence intensity in the cytoplasm (box in panel (**A**). The asterisks show the times when the laser was applied.

Next, we examined whether foreign molecules enter cells using this new laser poration method. When the laser beam was applied to single Cos-1 cells (African green monkey kidney cell line) in the presence of 10 μM FM1-43, a fluorescent lipid analogue in the external solution, the fluorescence intensity increased in the cytoplasm. The laser beam could be applied repeatedly to the same cells. During the second application, the fluorescence intensity increased twofold in the cytoplasm, suggesting that the laser poration quantitatively introduced a certain amount of foreign molecules into the cells (Fig. 3). Similarly, multiple introductions were possible in *Dictyostelium* cells (data not shown). The efficiency of successful introduction was 100% (n = 200). The laser power was much lower (1/10) than that used in the experiment demonstrated in Fig. 1. With this lower power, the carbon still remained; therefore, multiple applications of laser were possible.

**Figure 4:**
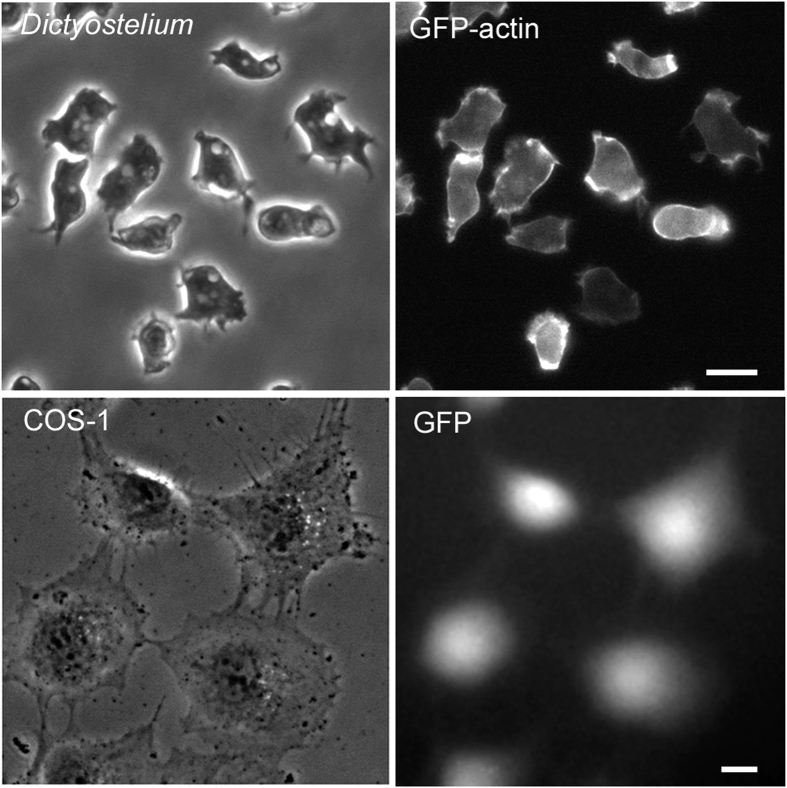
Transfection of DNA into *Dictyostelium* and Cos-1 cells. Laser poration was applied to *Dictyostelium* cells in the presence of a GFP-actin expression vector and Cos-1 cells in the presence of an EGFP expression vector. Both of the cell types were observed after one day using phase-contrast and fluorescence microscopy. Bars, 10 μm.

Next, we attempted to transfect DNA plasmids into cells. When the laser beam was applied to *Dictyostelium* cells in the presence of the GFP-actin expression vector, all of the applied cells showed fluorescence due to GFP-actin after a day (Fig. 4). The transfection efficiency achieved by this technique was always 100% (n = 25) without any loss of cell viability. Similarly, the transfection of an EGFP expression vector into Cos-1 cells was also successful (Fig. 4).

## Discussion

Most of the chemical methods, such as lipofection and others using any carriers, enable highly efficient transfection, sometimes up to 90%. However, the chemical information of these commercial transfection reagents is not supplied by the individual companies in many cases. The cells transfected using these chemicals cannot be readily used for therapeutic purposes for humans because the chemicals may remain in the transfected cells. By the same reason, virus-mediated transfection also cannot be used for human treatment. In addition, lipofection methods are not suitable for most primary isolated cells, blood cells, and neuronal cells. *Dictyostelium* cells also cannot be transfected by lipofection, though we have tried many types numerous times.

On the other hand, the cells transfected by the physical methods, such as microinjection or electroporation, can be readily used for research and even for therapeutic purposes. However, the transfection efficiency by electroporation is very low (usually 10^−7^–10^−4^). Microinjection is a reliable method but requires high skill especially for smaller cells and is time-consuming. Recently, directly poking the cell membrane with a nanoneedle under microscopy, which is usually used for atomic force microscopy, has been proven to generate pores in the cell membrane with an efficient transfer^6^, but this techniques is also time-consuming and requires a highly skilled practitioner.

Recently, combination methods using a laser and carriers have been invented. When a femtosecond laser beam is applied to cells in the presence of carbon nanoparticles and DNA, the laser-induced photoacoustic forces cause a transient permeabilization of the cellular membrane, resulting in the highly efficient delivery of DNA into the cells^7^. Alternatively, when gold nanoparticles are used instead of carbon nanoparticles, the laser-induced plasmonic effects on the gold nanoparticles cause the same effect^8^. However, both of these methods require that the cells are detached from the substratum and mixed with the DNA in suspension. The transfection efficiency and cell viability are also not very high.

The novel laser poration technique described here has several advantages. 1) This technique generates a pore only at the cell membrane without leaving any damage. 2) This method is time-saving, as the total operation time is only 5 min. 3) The minimum volume required is only 10 μl, including the cells and the external solution. Thereby, this method saves invaluable molecules. 4) Theoretically, this method is applicable to any cell. The introduction into single cells using microscopy is especially essential for rarely available, long-living and slow-growing cells such as neurons. 5) A lower power laser is sufficient to generate a pore in the cell membrane. Thus, the present device can be built at a lower cost. 6) Foreign molecules can be quantitatively introduced into cells by repeating the laser beam application. 7) Foreign molecules can be introduced at specific regions of the cell because the pore size is much smaller than the cell body. 8) Multiple species of foreign molecules can be simultaneously introduced into the same cell if these molecules are mixed in the external solution. 9) Cell communication between two adherent cells can be examined after introducing different foreign molecules into each cell. 10) All of the assigned cells can be transfected if the cells are assigned using an automatic stage. 11) This technique may also be applied to non-adherent cells if the coverslip is coated with adhesive substances such as polylysine.

In conclusion, this novel laser poration technique will provide enormous advantages to the introduction of foreign molecules into single cells under microscopy and contribute not only to research but also to applied biomedical fields, such as the establishment of iPS cells for regenerative medicine.

## Methods

### Cell preparation

*Dictyostelium* cells (AX2) were cultured in plastic dishes at 22 °C in HL5 medium (1.3% bacteriological peptone, 0.75% yeast extract, 85.5 mM D-glucose, 3.5 mM Na_2_HPO_4_. 12H_2_O, 3.5 mM KH_2_PO_4_, pH 6.3), as previously described^9^. The cells expressing GFP-lifeact were cultured in HL5 medium supplemented with 10 μg/ml G418 (Sigma). HL5 medium was exchanged with BSS (3 mM CaCl_2_, 10 mM KCl, 10 mM NaCl, 3 mM MES, pH 6.3), and the cells were incubated in the same solution for 3–5 h.

The Simian-virus-40-transformed African green monkey kidney cell line, COS-1, was maintained in glass-bottom dishes containing DMEM (Gibco BRL) supplemented with 10% FCS (Gibco BRL).

### Carbon coating

The surface of the coverslip of a glass-bottom chamber was coated with carbon by vapor deposition using a vacuum evaporator (JEOL, JEE-400). The thickness of the carbon layer was 20 nm. To make the surface hydrophilic, the surface of the carbon-coated coverslip was activated with a plasma treatment. The dish was sterilized with 70% ethanol and dried.

### Device Setup

A nanosecond-pulsed laser (FDSS532-Q, CryLas) beam was directed toward the sample by a dichroic mirror through an inverted fluorescence or total internal reflection fluorescence microscope (IX70, Olympus). The laser beam (wavelength, 532 nm) was operated at a 15 mW output power and pulse width of 1 nsec and attenuated to 1/300 by passing several neutral density filters. A 60× (PLAPON60XOTIRFM, Olympus, NA = 1.45) objective was used to focus the laser beam on the surface of the carbon-coat. When the laser beam was applied by properly adjusting the focus, the carbon coat was peeled off, appearing as a small white spot (Fig. 1B). The x-y position of the laser spot was fixed in the center of the microscopic field. The position of the cells was moved using a motorized x-y stage on the microscope. The duration of the laser beam application could be set at 8–500 msec and controlled by a shutter. All of the images were acquired by a cooled CCD camera (Orca ER, Hamamatsu Photonics) and processed with Image J software (http://rsbweb.nih.gov/ij/).

### Laser poration

*Dictyostelium* cells were cultured in the carbon-coated glass-bottom dish. After the medium was removed, a small aliquot (~10 μl) of 1 μM FM1-43 (Life Technologies) or 0.05 mg/ml of a GFP-actin expression vector^10^ was applied on the cells. Otherwise, the cell suspension was mixed with these molecules and then placed on the surface of the carbon-coated coverslip. After the cells settled down on the surface of the coverslip, a laser beam was applied at a small point on the cells for 10 msec. For the Cos-1 cells, the medium was exchanged with serum-free medium (OPTI-MEM1, Life Technologies) and then most of the medium was removed, leaving 10–20 μl of residual medium. Finally, 0.5 μl of a solution containing 0.5 mg/ml of an EGFP-expression vector was mixed. A laser beam was applied in the same way as for the *Dictyostelium* cells.

## Additional Information

**How to cite this article**: Yumura, S. A novel low-power laser-mediated transfer of foreign molecules into cells. *Sci. Rep.* **6**, 22055; doi: 10.1038/srep22055 (2016).
